# Circulating miR-28-5p is overexpressed in patients with sarcopenia despite long-term remission of Cushing’s syndrome: a pilot study

**DOI:** 10.3389/fendo.2024.1410080

**Published:** 2024-07-17

**Authors:** Marta Seco-Cervera, José Santiago Ibáñez-Cabellos, Federico V. Pallardo, José-Luis García-Giménez, Anna Aulinas, Luciana Martel-Duguech, Susan M. Webb, Elena Valassi

**Affiliations:** ^1^ Unit 733, Centre for Biomedical Network Research on Rare Diseases [CIBERER- Instituto de Salud Carlos III (ISCIII)], Madrid, Spain; ^2^ Mixed Unit for rare diseases INCLIVA-CIPF, INCLIVA Health Research Institute, Valencia, Spain; ^3^ Department of Physiology, Faculty of Medicine and Dentistry, University of Valencia, Valencia, Spain; ^4^ EpiDisease S.L., Scientific Park, University of Valencia, Paterna, Spain; ^5^ Department of Endocrinology, Hospital S Pau, Research Center for Pituitary Diseases, Institut de Recerca Sant Pau (IIB-Sant Pau), Barcelona, Spain; ^6^ CIBERER Unit 747, Instituto de Salud Carlos III, Madrid, Spain; ^7^ Department of Medicine, Universitat de Vic-Universitat Central de Catalunya, Vic, Spain; ^8^ Department of Medicine, Univ Autonoma Barcelona, Bellaterra, Spain; ^9^ Endocrinology and Nutrition Department, Germans Trias i Pujol Hospital and Research Institute, Badalona, Spain; ^10^ School of Medicine, Universitat Internacional de Catalunya (UIC), Sant Cugat del Vallès, Barcelona, Spain

**Keywords:** microRNA, miR-28-5p, myomiRs, sarcopenia, myopathy, Cushing syndrome

## Abstract

**Introduction:**

Patients with Cushing’s syndrome (CS) in remission show sustained fatigue, myopathy, and an increased prevalence of sarcopenia. The mechanisms that determine these persistent muscle problems are not well known. We aimed to identify circulating microRNAs (miRNAs) with differential expression that could be potential biomarkers for the diagnosis and/or prognosis in CS.

**Patients and methods:**

Thirty-six women in sustained remission for 13 ± 7 years (mean ± SD) from CS, with a median age (IQ range) of 51 (45.2–60) years and mean ± SD BMI of 27 ± 4 Kg/m^2^, and 36 matched healthy controls were investigated. In 7 patients sarcopenia was present according to the European Working Group on Sarcopenia in Older People (EWGSOP) criteria. Small RNA libraries were generated and indexed using a modified Illumina TruSeq small RNA-sequencing protocol. MiRNAs were identified in plasma using bioinformatic analysis, and validation was carried out using RT-qPCR. For the validation, Taqman probes were performed on QuantStudio 5 equipment (Applied Biosystems).

**Results:**

In a first discovery group using RNA-sequencing, plasma samples of 18 CS patients and 18 healthy subjects were investigated; circulating miR-28-5p, miR-495-3p and miR-654-5p were upregulated in CS patients as compared with controls (p<0.05). In a validation study of the 3 upregulated miRNAs in 36 patients and 26 controls, no differences were observed by RT-qPCR; however, the expression of circulating miR-28-5p was upregulated in CS patients with sarcopenia as compared with those without (AUC for fold-change in the ROC analysis, 0.798; p=0.0156). The optimized cut-off value for miR-28-5p to identify CS patients with sarcopenia was 3.80, which yielded a sensitivity of 86% and a specificity of 69%.

**Conclusion:**

MiR-28-5p, a muscle-specific microRNA involved in myotube proliferation and differentiation *in vivo*, may serve as an independent non-invasive biomarker for identifying CS patients at high-risk of sarcopenia despite biochemical remission.

## Introduction

Cushing’s syndrome (CS) develops because of endogenous hypercortisolism of pituitary or adrenal origin, and more rarely due to ectopic secretion of ACTH. It is a rare disease, which is treated by surgical excision of the originating lesion; drug therapy is also available if surgery is contraindicated, or unsuccessful, and pituitary irradiation is a possibility in recurrent or aggressive disease (1, 2). Over two thirds of patients treated for CS complain of muscle weakness, mainly affecting the pelvic girdle and lower limbs (1–3). In active disease, skeletal muscle mass is decreased in comparison to body mass index (BMI)‐matched controls (1, 4–6). Furthermore, it is known that steroids inhibit protein synthesis and enhance protein catabolism, with a consequent selective depletion of contractile proteins, myofibrillar degradation and atrophy of type IIa, fast‐twitch muscle fibers (4). Patients’ perception of muscle weakness despite endocrine “cure” is confirmed by poorer muscle performance despite unchanged muscle mass in women in remission, as compared with age-, and BMI-matched female controls (7). This muscle dysfunction is associated with increased intramuscular fatty infiltration and changes in muscle composition and architecture, which persist long term after remission of hypercortisolemia (7, 8). On the other hand, sarcopenia is currently considered by the European Working Group on Sarcopenia in Older People 2 -EWGSOP2- a muscle disease characterized by reduced muscle strength and function, but not necessarily associated with low muscle mass (9). We have previously shown that sarcopenia is increased in “cured” CS patients compared to matched controls and impacts considerably on patients perceived health related quality of life (10).

The mechanisms that determine muscle dysfunction after chronic exposure to hypercortisolemia are not well understood. In recent years, ample evidence has been published on the prominent role of microRNAs (miRNAs) in regulating skeletal muscle plasticity and functionality as well as fiber-type specificity and metabolic properties (11). MiRNAs are small non-coding RNA molecules involved in the post-transcriptional regulation of gene expression by binding to their target messenger RNAs (mRNAs). Some miRNAs are ubiquitously expressed in tissue, while others are tissue-specific or tissue enriched; miRNAs can also be expressed in biological fluids such as serum, saliva or urine, where they are resistant to degradation by RNAases (12). Skeletal muscle-specific miRNAs (“myomiRNAs”) are involved in myoblast proliferation, differentiation and regeneration, and changes in their circulating levels have been described in several conditions associated with abnormal muscle organization and function, including specific muscle diseases and age-related sarcopenia and frailty (13–16). Moreover, myomiRNAs have been suggested as new biomarkers of physiological and pathological muscle processes (14, 17–22). Furthermore, in a C2C12 mouse myocyte model, hydrocortisone induced overexpression of miR-133a-3p and atrophic signals, also observed in patients with active CS, leading the authors to suggest that this myomiRNA might be a potential biomarker to discriminate between healthy controls and patients with CS (23). Given that prior exposure to hypercortisolism in the context of CS determines residual musculoskeletal morbidity, we aimed at evaluating differentially expressed miRNAs in plasma of patients with CS in remission, and whether they are related to sarcopenia.

## Patients and methods

### Patients

Thirty-six women with CS in remission and 36 age- and BMI-matched female controls were included. Diagnosis of CS was reached after evaluation of clinical, biochemical, and radiological data, based on international guidelines (24) as previously reported (7). Seven patients had sarcopenia according to the definition of the European Working Group on Sarcopenia in Older People (EWGSOP), as previously described (10). Inclusion criteria were age up to 65 years and in remission a minimum of 3 years. Twenty‐eight patients had Cushing’s disease (CD) due to a pituitary microadenoma (n = 25) or a macroadenoma (n = 3) and 8 had an adrenal adenoma. The median (interquartile range) duration of hypercortisolism (defined as the time since initial symptoms, as referred by patients, until final diagnosis of CS) was 33 (12–36) months.

In the 28 CD patients transsphenoidal surgery was performed a median of 154 (117) months before the study; 30 patients (83%) received preoperative treatment with steroidogenesis inhibitors to control clinical symptoms of hypercortisolism; 7 (19%) had undergone radiotherapy a median of 142 (72–180) months after unsuccessful surgery (n = 1) or relapse (n = 6). Adrenalectomy was performed in the 8 patients with an adrenal adenoma a median of 100 months (89–38) months before the study. Mean (± SD) time of remission (time since diagnostic confirmation of remission to study entry) was 13 ± 7 years (median, 13 [6–14] and range, 1–17 years]. Remission was considered if adrenal insufficiency (basal morning cortisol <171 nmol/L [<6.2 μg/dL] and/or undetectable 24‐h free urinary cortisol) was observed, or morning cortisol suppressed <50 nmol/L (<1.8 μg/dL) after an overnight 1 mg dexamethasone suppression test. Hydrocortisone (HC) replacement (10 to 20 mg/day) was required for a median of 19 (0–24) months after surgery in 30 patients (83%); median time free from HC replacement was 84 (36–140) months. At study entry, 3 patients were still on HC substitution therapy at a stable dose of 20 mg/day (mean ± SD duration of treatment 148 ± 35 months), 3 had growth hormone deficiency (2 treated with recombinant human GH for a mean ± SD duration of 38 ± 42 months); 5 were hypothyroid (3 due to TSH deficiency and 2 due to primary hypothyroidism), on stable replacement doses (65 ± 22 μg/day) of L‐thyroxine (mean ± SD duration of treatment 127 ± 32 months). Fifty-eight percent (n=21) were postmenopausal (mean ± SD duration of menopause of 96 ± 61 months). No patient had postoperative gonadotropin deficiency nor was treated with oestrogen/progesterone hormone replacement. Exclusion criteria included being older than 65 years, active disease, inflammatory disorders, diabetes mellitus, kidney, liver or neurological dysfunctions, malignancies, documented physical disability or motility limitations and treatment with local or systemic glucocorticoids during the previous year.

For each of the 36 CS patients, a female blood donor matched for age, BMI, menopausal status and degree of physical activity was identified and recruited after consent to participate (**Table 1**). Clinical data on disease characteristics and physical examination were collected as well as fasting blood samples in EDTA tubes for RNA analysis.

**Table 1:** Age and Body Mass Index (BMI) of the study groups (mean ± SD).

DISCOVERY GROUP	CONTROLS (n=18)	CS (n=18)	CONTROLS Vs. CS	WITHOUT SARCOPENIA (n=12)	WITH SARCOPENIA (n=6)	WITHOUT Vs. WITH SARCOPENIA
Age years	53.1 ± 9.1	56 ± 6	P = 0.28	54.9 ± 6.2	58.2 ± 5.5	P = 0.3
BMI Kg/m^2^	27.5 ± 3.8	27.5 ± 3.6	P = 0.94	27.3 ± 3.3	27.3 ± 3.9	P = 0.79

VALIDATION GROUP	CONTROL (n=26)	CS (n=36)	CONTROLS Vs. CS	WITHOUT SARCOPENIA (n=29)	WITH SARCOPENIA (n=7)	WITHOUT Vs. WITH SARCOPENIA
Age years	50.3 ± 12.4	49.9 ± 11.6	P = 0.88	48.4 ± 12	56.1 ± 7.3	P = 0.16
BMI Kg/m^2^	27 ± 4.1	26.6 ± 3.7	P = 0.89	26.1 ± 3.7	28.7 ± 3.3	P = 0.1

The discovery group was formed by 18 patients with Cushing’s syndrome (CS) of which 6 had sarcopenia, and 18 controls; the validation group included 36 CS patients, of which 7 had sarcopenia, and 26 controls. No significant differences (p-value <0.05) were observed between groups.

The study was approved by the ethics committee of our institution (IIB‐CEIC), and all subjects gave full, written consent.

### RNA extraction and quantification

Blood samples from CS patients and controls were centrifuged at 2500 rpm for 10 minutes to separate the plasma, followed by a second centrifugation at 16000g and 4 °C for 10 minutes. Absorbance at 414nm and 385nm were measured in NanoDrop™ One Microvolume UV-Vis Spectrophotometer (Thermo Scientific, DE, USA) to check the quality of plasma and discard those samples that had A414/385 >3, indicative of haemolysis (n=10 controls). Plasma samples were stored at −80°C until RNA extraction. Cell-free total RNA (including miRNAs) was extracted from 400 µL of plasma using the miRNeasy Serum/Plasma kit (Qiagen, CA, USA), following the manufacturer’s protocol. The free RNA was eluted with 25 µL of RNAse-free water. The concentration of cell-free total RNA (including miRNAs) was quantified using NanoDrop™ One Microvolume UV-Vis Spectrophotometer (Thermo Scientific, DE, USA).

### Library preparation and small RNA-sequencing of the discovery group

To perform small RNA libraries, we selected a discovery group of samples from 18 CS patients (6 with sarcopenia) and 18 healthy subjects (**Table 1**). The libraries were generated and indexed using a modified Illumina TruSeq small RNA protocol. In this modified protocol the libraries were size selected (range 90-170pb) using the Blue Pippin instrument (Sage Science, Beverly, MA, USA). A positive RNA control was included (Thermo Fisher Scientific Human Brain Total RNA catalogue #AM7962). SmallRNA-sequencing (smallRNA-seq) was performed by single-end sequencing on Illumina NextSeq550 platform on High Output 1x50pb RUN (NextSeq 500/550 High Output v2 75 cycles kit, FC-404-2005).

### Differential expression analysis of the discovery group

The first step was to assess the quality of the Illumina raw sequences and trimming to remove sequencing adapters and low-quality bases. Thereafter, sequences were mapped against the human GRCh38 reference sequence, taken from Ensembl. After that, the intersection between the aligned position of reads and the miRNA coordinates taken from miRBase v22 was performed. The alignment and quantification steps were performed using Subread and Rsubread Packages.

### Prediction of miRNA targets and over-representation analysis of the discovery group

Firstly, we accessed miRanda and DIANA-microT-CDS from the DIANA web server (25), to predict messenger RNAs (mRNAs) which are potential targets of miRNAs. Moreover, we used TarBase v7.0 to identify those mRNAs which have been already validated as targets of our miRNA candidates. An Over-Representation Analysis (ORA) was carried out, using Gene Ontology (GO) terms and the Kyoto Encyclopaedia of Genes and Genomes (KEGG), using as default parameters experimentally supported interactions from DIANA TarBase v.7.0 with a p-value threshold of 0.001, and a microT threshold of 0.8. To reduce the number of false positive miRNA targets, we applied a false discovery rate (FDR) correction to the selected KEGG pathways. The algorithm used in this analysis was a one-tailed Fisher’s exact test.

### Validation by Real-time qPCR of miRNA signatures from plasma in a validation group

To confirm the data from the sequencing experiments new samples from CS and healthy controls were included. Specifically, the 18 samples of CS patients and the 18 of healthy controls of the discovery group were used and a further 18 CS patient samples and 8 healthy controls were added, resulting in a total group of 36 patients with CS and 26 controls.

Reverse transcription reactions were performed using the TaqMan miRNA Reverse Transcription kit and miRNA-specific stem-loop primers (Part No. 4366597, Applied Biosystems. Inc, CA; USA) and 100 ng of input cell-free RNA in a 20 µL RT reaction. Real-time PCR reactions were performed in triplicate, in scaled-down 10 µL reaction volumes using 5 µL TaqMan 2x Universal PCR Master Mix (Applied Biosystems. Inc, CA; USA) with No UNG, 0.5 µL TaqMan Small RNA assay (20x) (Applied Biosystems. Inc, CA; USA), miR-28-5p (000411), miR-495-3p (001663), miR-654-5p (001611)], 3.5 µL of nuclease free water and 1 µL of RT product. Real-time PCR was carried out on an Applied BioSystems QuantStudio 5 (Applied Biosystems. Inc, CA; USA) programmed as follows: 50°C for 2 minutes, 95°C for 10 minutes followed by 45 cycles of 95°C for 15 seconds and 60°C for 1 minute. We used miR-191-5p (002299), one of the most stable miRNAs in terms of read counts, as previously described as an endogenous control (26–28) to normalize the expression in plasma samples. All the fold-change data were obtained using the delta-delta cycle threshold (Ct) method (2^-ΔΔCT^) (29).

### Validation of miRNAs as biomarkers

To assess differences between patients and controls and between patients with and without sarcopenia, Mann-Witney tests to compare miRNA fold-change values as a continuous variable in the 3 different groups were performed (healthy controls, CS with sarcopenia and CS without sarcopenia).

The miRNA diagnostic test utility was validated by a ROC curve analysis, measuring the area under the curve, diagnostic sensitivity and specificity, and positive and negative predictive values. Optimal cut-off points were determined by highest sensitivity plus specificity and efficiency values.

P-values below 0.05 were considered significant. Data analysis was performed using Graphpad Prism 8.0 (GraphPad Software, CA, USA).

## Results

### Clinical characteristics of Cushing’s syndrome patients and controls

The characteristics of the study populations are shown in **Table 1**. CS patients had a median age (interquartile range) of 51 (45.2–60) years and a mean (± SD) BMI of 27 ± 4 Kg/m2] and did not differ from the healthy controls; both groups did not differ as far as other clinical features (including time since menopause, and duration of remission, since diagnostic confirmation), as previously described (10). For the discovery group, 18 patients with CS and 18 healthy controls were investigated; 6 of the CS patients had sarcopenia. For the validation study there were 36 CS patients of which 7 had sarcopenia and 26 healthy controls.

### Identification of differentially expressed miRNAs by small RNA-sequencing

**Figure 1:**
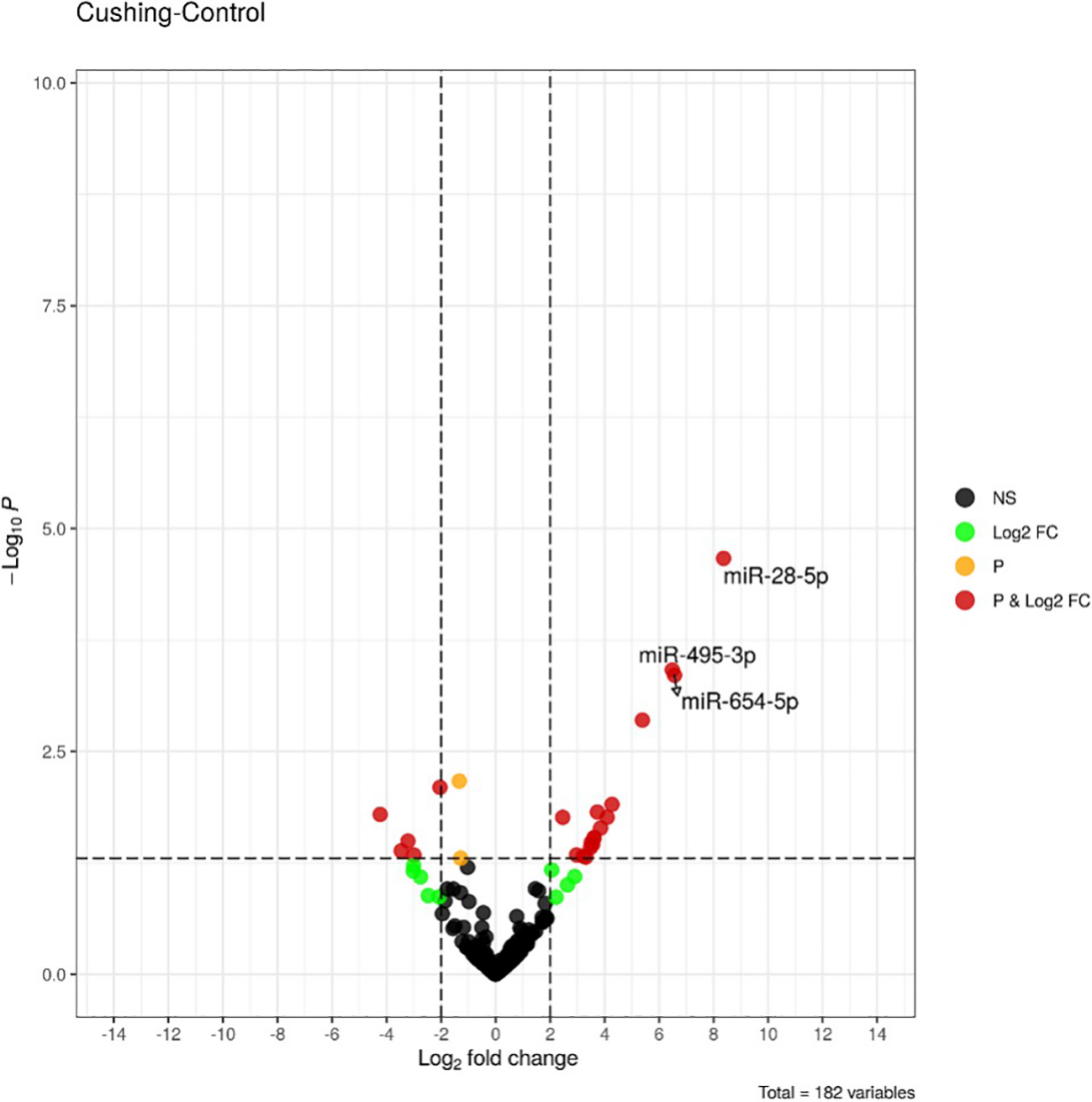
Volcano-plot of differentially expressed miRNAs between healthy controls (n = 18) and Cushing’s syndrome patients (n = 18) in the discovery group. Vertical lines indicate the threshold for a relative expression fold change (FC) of above 2 or below 2-fold compared to controls. The horizontal line represents the threshold of a 0.05 p-value. BLACK DOTS, NS: miRNAs with no significant change; GREEN DOTS, Log2FC: miRNAs with Log2 fold change > 2 in absolute values; ORANGE DOTS, P: miRNAs with a p-value < 0.05; RED DOTS, P&Log2FC: miRNAs with a p-value < 0.05 and a Log2 fold change > 2 in absolute values. miRNAs with false discovery rate (FDR) less <0.05 are labelled.

The expression analysis of miRNAs in the 18 patients with CS compared to the 18 healthy controls of the discovery group using small RNA-sequencing showed differential expression of 3 miRNAs, namely miR-28-5p, miR-495-3p, and miR-654-5p (false discovery rate < 0.05). As shown in **Figure 1**, **Supplementary Table 1** all these miRNAs were upregulated in patients compared to controls.

### Enrichment analysis of miRNA targets and pathways in the context of Cushing’s syndrome

**Figure 2:**
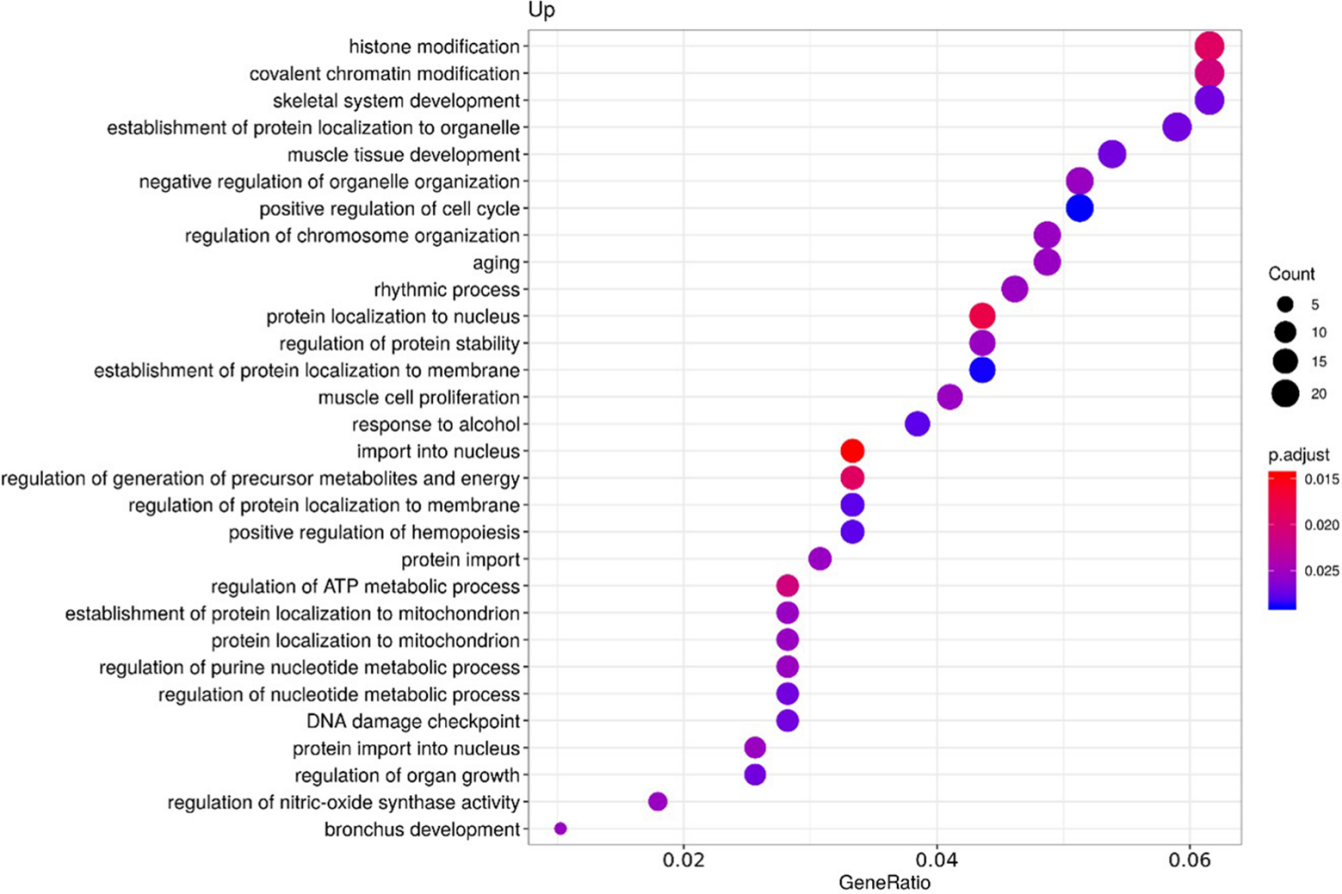
Functional enrichment by Over-representation analysis (ORA) using Gene Ontology (GO) terms. GO terms are classified according to the ratio of the number of target genes for each miRNA included in each GO term from the total of genes included in the GO term (Gene ratio), the number of counts per million for each miRNA in each GO term (Count) and the p-value adjusted by the Fisher test (p. adjust).

**Figure 3:**
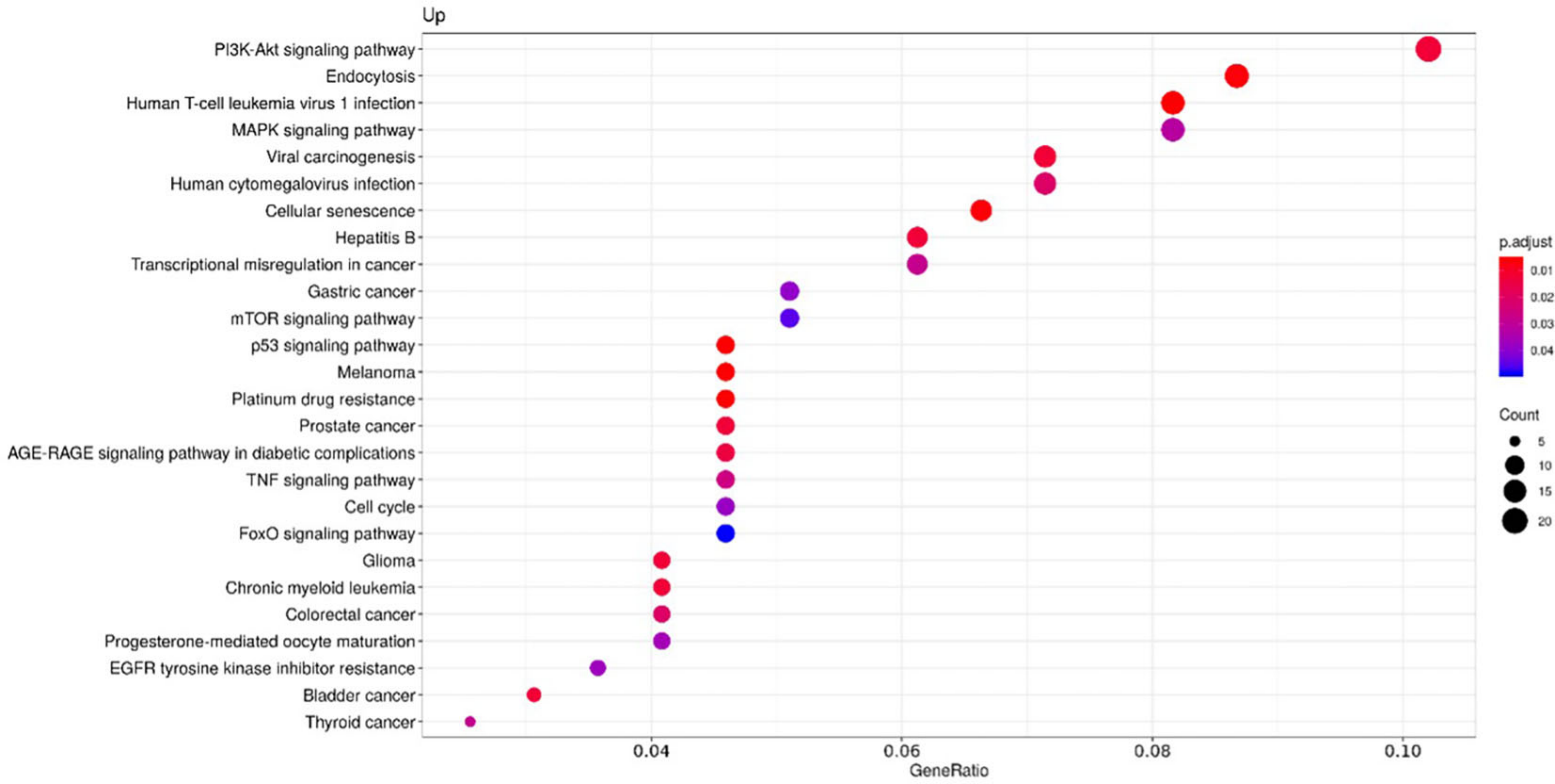
Functional enrichment by Over-representation analysis (ORA) using the Kyoto Encyclopaedia of Genes and Genomes (KEGG) pathways. KEGG pathways are classified according to the ratio of the number of target genes for each miRNA included in each pathway from the total of genes of the pathway (Gene ratio), the number of counts per million for each miRNA in each pathway (Count) and the p-value adjusted by the Fisher test (p. adjust).

To clarify the role of the identified upregulated miRNAs in CS, we analyzed biochemical networks that were regulated by these 3 miRNAs. After ORA analysis, using GO terms and the KEGG pathways significantly regulated by these altered miRNAs, we found a total of 98 GO terms with a p-value of less than 0.05, 81 corresponding to biological processes terms, 11 corresponding to cellular component terms, and 6 corresponding to molecular functions (**Supplementary Table 2**). The most significant biological processes terms are shown in **Figure 2**. These include skeletal system development, aging, and muscle cell proliferation. The KEGG analysis, showed 26 pathways with an FDR of less than 0.05 (**Supplementary Table 3**). The most significant pathways observed, considering the physiopathology of CS, are the MAPK signaling pathway, cellular senescence, and Tumor necrosis factor α (TNFα) signaling pathway (**Figure 3**).

### Validation of the differentially expressed miRNAs by RT-qPCR

**Figure 4:**
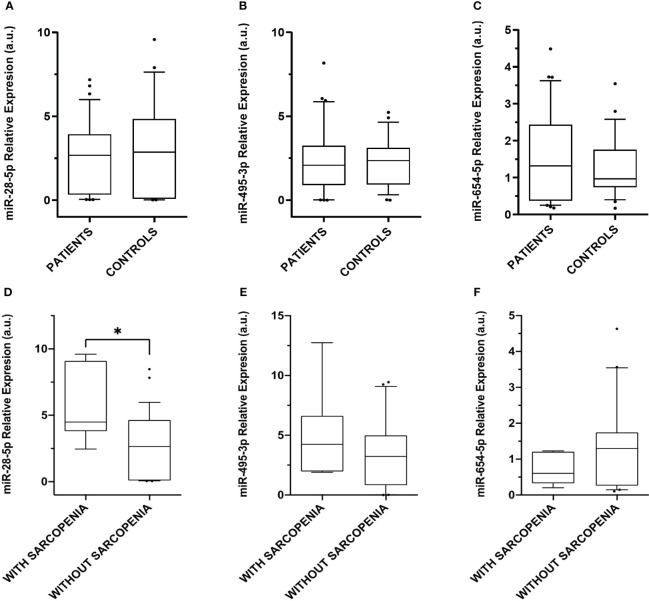
Relative expression levels of the miRNAs differentially expressed in plasma. Box plot levels of **(A)** miR-28-5p (p-value = 0.49); **(B)** miR-495-3p (p-value = 0.88), and **(C)** miR-654-5p (p-value = 0.47) in 36 CS patients compared to 26 controls; **(D)** miR-28-5p (* p-value = 0.01); **(E)** miR-495-3p (p-value = 0.43), and **(F)** miR-654-5p (p-value = 0.16) in CS patients without sarcopenia (n = 29) and CS with sarcopenia (n = 7). Expression levels of the miRNAs were normalized to miR-191-5p. The lines inside the boxes denote the medians. The boxes mark the interval between the 25th and 75th percentiles. The whiskers denote the interval between the 10th and 90th percentiles. Filled circles indicate data points outside the 10th and 90th percentiles. Differences were determined using Mann-Whitney tests. P-values were two-tailed and significant if < 0.05.

The 3 differentially expressed miRNAs detected by smallRNA-seq were validated by RT-qPCR, in 36 CS patients and 26 controls. The relative expression levels for each miRNA were calculated in all the samples analyzed by smallRNA-seq and RT-qPCR, respectively. No differences in plasma miRNAs present in CS patients and controls were observed, in contrast with the results obtained by smallRNA-seq in the discovery phase (**Figures 4A–C**).

### Phenotypic characterization of Cushing’s syndrome patients with and without sarcopenia according to miRNA expression

Due to the absence of significant differences observed in the validation assay by RT-qPCR, we evaluated the results in a second approach. Considering the results of the enrichment analysis that point out the relevance of these miRNAs in muscle cell proliferation and aging, we analyzed the expression of the 3 upregulated plasma miRNAs in different clinical subgroups, namely 7 CS patients with sarcopenia and 29 without sarcopenia, aimed at identifying specific miRNA signatures. Although no differences in miR-495-3p and miR-654-5p expression were observed, miR-28-5p was upregulated in the 7 CS patients with sarcopenia when compared with those without sarcopenia (**Figures 4D–F**).

### miR-28-5p as a phenotypic biomarker of sarcopenia in Cushing’s syndrome

**Figure 5:**
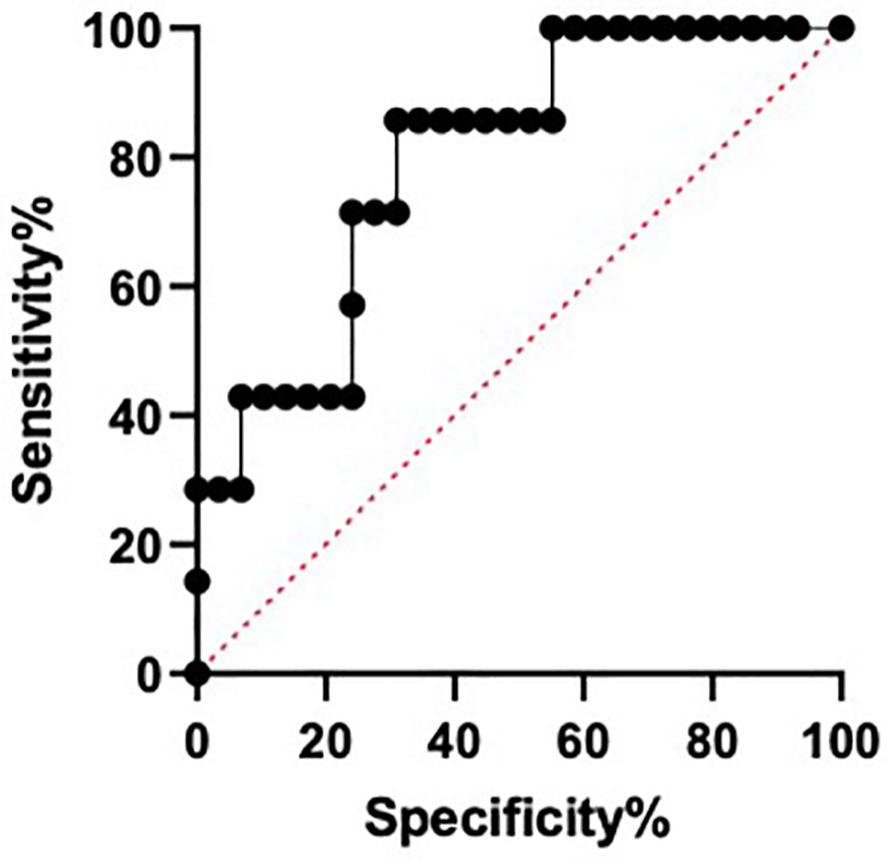
Area under the curve of receiver operating characteristic (ROC) for miR-28-5p as a possible biomarker of sarcopenia in Cushing´s syndrome.

Given the increase of miR-28-5p in CS patients with sarcopenia, its use as a possible biomarker of sarcopenia was evaluated. A ROC curve identified differences between the miR-28-5p fold change in CS patients with and without sarcopenia, with an AUC of 0.7980 (p=0.0156) and an optimal cut- off value for the fold change of 3.80, with a sensitivity of 86% and a specificity of 69% (**Figure 5**).

## Discussion

In this pilot study, we have found that patients with CS in remission and sarcopenia have higher circulating levels of miR-28-5p, a muscle-specific microRNA involved in myotube proliferation and differentiation, as compared with their counterparts without sarcopenia. A frequent complaint in patients diagnosed with CS is that despite remission, they suffer from sustained fatigue and myopathy, mainly affecting the pelvic girdle and lower limbs (1–3). The mechanisms that determine these persistent muscle problems are not well understood. Our findings suggest that plasma miR-28-5p may be a minimally invasive biomarker to identify those patients with prior CS who maintain a high-risk of sarcopenia despite normalization of cortisol levels and may contribute to discover physiological and molecular pathways underlying this disease.

Although in the first discovery approach using smallRNA-sequencing we identified an overrepresentation of 3 plasma miRNAs (miR-28-5p, miR-495-3p, and miR-654-5p) in CS patients compared to matched healthy control subjects, these results were not confirmed by RT-qPCR in the validation phase, where no differences in the expression of these 3 miRNAs were observed when comparing the whole group of CS patients with controls. The reason for this discrepancy is currently unclear; miRNA expression is known to be highly variable between individuals and is often age-dependent (30). Additionally, myomiRNAs function in a complex fashion in muscle tissues, potentially ‘buffering’ against physiological and pathological changes to maintain homeostasis. They are thought to target multiple gene transcripts in the same pathway to ensure a biological outcome. They may also display cooperativity and redundancy of different miRNAs by synergistically targeting the same transcript (31). Variations in the differential expression of miRNAs may depend on the methodology used (i.e., smallRNAseq or RT-qPCR), given the different normalization approach used to calculate the relative expression of miRNAs for each method. In this regard, small RNA-sequencing uses total counts of reads for each miRNA and the relative expression is calculated by normalizing from the whole counts obtained, while in RT-qPCR relative expression is calculated using an endogenous control to normalize relative expression of CS patients compared to the control subjects. Although it would be interesting to investigate other subgroups of patients with CS, the rarity of this disease prevents the study of large numbers of individuals to further explain the results obtained by RT-qPCR.

Since sequencing did not disclose any differences in the 3 miRNAs analyzed, another approach was adopted, based on the evidence from our enrichment analysis and the literature, by linking these miRNAs to sarcopenia, and evaluating the expression of miRNAs in CS patients with or without sarcopenia. With this approach overexpression of miR-28-5p in serum of patients in long-term remission of CS who suffer from sarcopenia as persistent morbidity was evidenced, compared to CS patients without this muscle dysfunction.

Changes in the expression of several members of the miR-28 family during muscle cell differentiation (32, 33) and in response to stress and exercise (22, 33, 34) suggest that they contribute to the control of muscle cell proliferation, tissue development, muscle regeneration and homeostasis (21, 35–38). Furthermore, the expression of skeletal muscle N-myc downstream-regulated gene 2 (NDRG2) protein is increased during muscle differentiation and development both *in vitro* and *in vivo* (39, 40), promoting myoblast proliferation (31). Overexpression of miR-23a, -23b and -28 in skeletal muscle cells of mice were shown to co-regulate mouse Ndrg2 in the presence of dexamethasone (31). These specific *in vitro* stress conditions, namely in the presence of a synthetic glucocorticoid like dexamethasone, represents a non-physiological condition for the regulation of Ndrg2. However, it could be compared to the chronic exposure to hypercortisolism typical of CS; the fact that in those CS patients with sarcopenia, i.e., with most severe permanent muscle dysfunction, miR-28 was also upregulated, would suggest that both may be linked. Whether miR-28 acts on its own or cooperatively with other miRNAs under similar stress conditions *in vivo*, targeting Ndrg2 is still unclear, as well as if it could represent a potential therapeutic target in catabolic muscle wasting conditions (31).

Further evidence of a relationship between skeletal muscle miR-28 and muscle development has been reported; namely downregulation of miR-28 was observed after aerobic exercise training for 6 weeks in healthy individuals - which will enhance muscle development (41, 42) -, as well as in goat kids, where downregulation of miR-28 promoted myoblast proliferation and differentiation (39). An interesting point for miR-28-5p is its possible implication in aging and myopathy. One target of this miRNA is nuclear factor erythroid 2-related factor 2 (NRF2). Huang et al. demonstrated that a deficiency of Nrf2 in a mouse model was associated with increased frailty and sarcopenia during aging, by altering mitochondrial biogenesis and dynamics (43).

On evaluating the relevant GO terms identified in our enrichment analysis using the KEGG pathways, muscle tissue development and muscle cell proliferation were identified. Both pathways contain common genes regulated by miRNAs, like the *MEF2D* gene, which encodes for the Myocyte-specific enhancer factor 2D that is targeted by miR-28-5p and miR-654-5p; or the *ZBTB18* gene that encodes for Zinc finger and BTB domain-containing protein 18, a transcriptional repressor that regulates myogenesis (44), also targeted by miR-28-5p. These terms are important in the context of CS since muscle wasting and sarcopenia are common complaints in this disease (10, 45). Both miR-654-5p and miR-28-5p have similar effects on vascular smooth muscle cell proliferation and migration, namely an inhibitory effect if they are upregulated and a stimulatory effect when they are downregulated, via the FOXO pathway (46) or by directly targeting ADAMTS-7 (a disintegrin and metalloproteinase with thrombospondin motifs-7) (47). But to the best of our knowledge, no reports on miR-654-5p related to skeletal muscle have been described to date.

Considering the pathophysiology of CS, the MAPK/ERK signaling pathway is a further identified KEGG term; interestingly, it has also been described to be involved in sarcopenia related to aging (32–34). In fact, miR-28-5p targets two genes included in this pathway, namely Insulin-like growth factor-I (IGF-I) and serum response factor (SRF), both related to sarcopenia and that play a role as regulators of muscle atrophy (21, 35–38). Cellular senescence is another relevant pathway identified, since CS has been associated to premature aging and telomere length shortening, as well as with dyslipidemia, osteoporosis, glucose intolerance and elevation of chronic inflammatory markers (48, 49). Additionally, the tumor necrosis factor α (TNFα) signaling pathway was also overrepresented. We have shown that plasma soluble TNFα receptors (sTNF-R1 and sTNF-R2) are increased in long-term cured CS patients (50) and its overexpression may play a significant role in residual complications like myopathy and muscle atrophy (51). One of the genes of the TNFα signaling pathway is C-C motif chemokine ligand 2 (CCL2) a promotor of myogenesis via the AKT/mTOR signaling pathway, recently associated to active CS (23). They report overexpression of miR-133a-3p in both mouse myocytes exposed *in vitro* to hydrocortisone and serum of patients with active CS (27 patients with CD including 6 males and 10 with adrenal CS, including 2 males), where it was higher than in controls, leading them to suggest that circulating miR-133a-3p could be a promising biomarker of hypercortisolism. In the present study, which differed in the sense that patients were in long-term remission of CS and included both genders, this myomiR was not found to be differentially expressed. Further investigations will be necessary to better understand the role of these myomiRs in CS, both active and in remission.

An attempt to use circulating microRNA expression to identify markers of pituitary CD versus adrenal cortisol-producing adenoma reported upregulation of miR-182-5p using qPCR in the CD group but no differences between controls and the whole CS cohort from the German Cushing’s registry (52).

Among the limitations of the present pilot study are its cross-sectional design, which prevents from inferring causality from our data. Additionally, the small groups of patients is practically unavoidable in a rare disease like CS and the limitation of the number of patients may impact directly in the differences observed in the expression of miRNAs detected by smallRNA-seq and RT-qPCR, since their results depend on the different methods used for normalizing the relative expression of miRNAs. Only women were included given the greater prevalence of CS in the female gender (above 70%) which precluded including a sufficient number of men. However, a strength of our study is the detailed screening of sarcopenia in the CS patients with strict criteria, using both physical performance indices and imaging parameters, as defined by the European Working group on Sarcopenia in Older People 2 -EWGSOP2- (9), considered as the gold standard to measure the actual ratio of muscle and fat.

In summary, we have identified free plasma miR-28-5p as a possible non-invasive potential biomarker of the presence of sarcopenia as a long-term persistent consequence of CS, despite attaining remission of hypercortisolemia. Identifying these patients with sarcopenia to offer an adapted exercise program would very likely benefit their long-term prognosis, since improving muscle weakness can prevent falls, as well as benefitting perceived health and quality of life (10, 53). Future studies should confirm whether patients with sarcopenia of another origin (not related to CS) also exhibit this upregulation of miR-28-5p.

## Data Availability

The datasets presented in this study can be found in online repositories. The names of the repository/repositories and accession number(s) can be found below. GEO (Gene Expression Omnibus) repository, accession number GSE271649.

## Supplementary material

Supplementary Table 1 Identification of differentially expressed miRNAs by small RNA-sequencing.

Supplementary Table 2 Over-representation analysis using Gene Ontology terms to identify pathways significantly regulated by the identified miRNAs (miR-28-5p, miR-495-3p and miR-654-5p).

Supplementary Table 3 Over-representation analysis using the Kyoto Encyclopaedia of Genes and Genomes to identify pathways significantly regulated by the identified miRNAs (miR-28-5p, miR-495-3p and miR-654-5p).
