# Corrigendum: Circulating miR-28-5p is overexpressed in patients with sarcopenia despite long-term remission of Cushing’s syndrome: a pilot study

**DOI:** 10.3389/fendo.2025.1587462

**Published:** 2025-06-19

**Authors:** Marta Seco-Cervera, José Santiago Ibáñez-Cabellos, Federico V. Pallardo, José-Luis García-Giménez, Anna Aulinas, Luciana Martel-Duguech, Susan M. Webb, Elena Valassi

**Affiliations:** ^1^ Unit 733, Centre for Biomedical Network Research on Rare Diseases [CIBERER- Instituto de Salud Carlos III (ISCIII)], Madrid, Spain; ^2^ Mixed Unit for rare diseases INCLIVA-CIPF, INCLIVA Health Research Institute, Valencia, Spain; ^3^ Department of Physiology, Faculty of Medicine and Dentistry, University of Valencia, Valencia, Spain; ^4^ EpiDisease S.L., Scientific Park, University of Valencia, Paterna, Spain; ^5^ Department of Endocrinology, Hospital S Pau, Research Center for Pituitary Diseases, Institut de Recerca Sant Pau (IIB-Sant Pau), Barcelona, Spain; ^6^ CIBERER Unit 747, Instituto de Salud Carlos III, Madrid, Spain; ^7^ Department of Medicine, Universitat de Vic-Universitat Central de Catalunya, Vic, Spain; ^8^ Department of Medicine, Univ Autonoma Barcelona, Bellaterra, Spain; ^9^ Endocrinology and Nutrition Department, Germans Trias i Pujol Hospital and Research Institute, Badalona, Spain; ^10^ School of Medicine, Universitat Internacional de Catalunya (UIC), Sant Cugat del Vallès, Barcelona, Spain

**Keywords:** microRNA, miR-28-5p, myomiRs, sarcopenia, myopathy, Cushing syndrome

In the published article, there was an error in the **Funding** statement. The funding statement was displayed as “The author(s) declare that no financial support was received for the research, authorship, and/or publication of this article.”. The correct Funding statement appears below.

“The author(s) declare that financial support was received for the research, authorship, and/or publication of this article. This work was supported by a grant from the Instituto de Salud Carlos III (FIS PI21/01223; PI, Elena Valassi), European Regional Development Fund (FEDER) funds.”

The authors apologize for this error and state that this does not change the scientific conclusions of the article in any way. The original article has been updated.

